# Aortic Pseudoaneurysm Mimicking a Pulmonary Neoplasm in an 87-Year-Old Patient: The Challenge of the “Great Mimicker” in Emergency Medicine

**DOI:** 10.7759/cureus.113512

**Published:** 2026-07-28

**Authors:** Johana A Sisa Rodríguez, Iván L Rosso, Maria A Pérez Huelgas, Jason J Sisa Rodríguez, Wilmer Aponte

**Affiliations:** 1 Radiology, Universidad Nacional de Colombia, Bogotá, COL

**Keywords:** aortic pseudoaneurysm, differential diagnosis, endovascular procedures, frail elderly, lung neoplasm, penetrating aortic ulcer

## Abstract

Acute aortic syndrome (AAS) is a cardiovascular emergency associated with high mortality and often presents a diagnostic challenge, particularly in geriatric patients. We report the case of an 87-year-old woman who presented with acute chest pain, elevated biomarkers (troponin T and D-dimer), and an incidental finding on computed tomography (CT) of a mass in the left pulmonary apex. Initially suspected to represent a lung neoplasm because of associated constitutional symptoms, multidisciplinary review and dedicated aortic imaging subsequently revealed a pseudoaneurysm arising from a penetrating aortic ulcer of the distal aortic arch. The patient was successfully treated with straightforward thoracic endovascular aortic repair (TEVAR), without the need for supra-aortic branch coverage or adjunctive revascularization procedures, as the lesion was located distal to the origin of the supra-aortic branches. This case highlights the importance of considering AAS in the differential diagnosis of mediastinal and pulmonary masses in elderly patients.

## Introduction

Acute aortic syndrome (AAS) encompasses a group of highly lethal conditions, including aortic dissection, intramural hematoma, and penetrating aortic ulcer (PAU) [1,2]. These conditions share pathophysiological mechanisms related to aortic wall injury and typically manifest with severe acute chest pain [1,3]. However, the clinical presentation can be variable and may occasionally mimic pulmonary or mediastinal diseases, thereby delaying timely diagnosis and treatment [4,5].

PAU represents a less frequent form of AAS. It occurs when an ulcerated atherosclerotic plaque erodes the internal elastic lamina and penetrates the aortic media, potentially progressing to intramural hematoma, pseudoaneurysm, or contained rupture [2,6]. Lesions located in the aortic arch or descending thoracic aorta can produce radiologic findings suggestive of a pulmonary or mediastinal mass, particularly in the presence of periaortic inflammation or an adjacent hematoma [4,5].

We present the case of an elderly woman with chest pain and an initial finding of a left apical lung mass suggestive of a pulmonary neoplasm, in whom a contained thoracic pseudoaneurysm secondary to a PAU was subsequently confirmed [4].

## Case presentation

An 87-year-old woman with no known prior medical history presented to the emergency department with a five-hour history of oppressive retrosternal chest pain radiating to both shoulders and associated with dyspnea. On admission, she was hemodynamically stable (blood pressure 125/45 mmHg, heart rate 75 bpm) but continued to report severe chest pain. Electrocardiography showed sinus rhythm without signs of acute ischemia.

Initial laboratory studies performed at the referring institution revealed markedly elevated biomarkers, including troponin T (22.64 ng/mL; reference value <0.014 ng/mL) and D-dimer (8,802 ng/mL; reference value <500 ng/mL). Complete blood count demonstrated leukocytosis (12,670 cells/µL; reference range 4,000-10,000 cells/µL) with neutrophilia and anemia (hemoglobin 8.3 g/dL; reference range 12-16 g/dL). Repeat laboratory testing at our institution showed troponin I levels of 0.012 ng/mL and 0.010 ng/mL (reference value <0.056 ng/mL), as well as a D-dimer level of 4,491 ng/mL (Table 1). Given the suspicion of acute coronary syndrome (ACS) or pulmonary embolism (PE), anti-ischemic therapy with aspirin, clopidogrel, and atorvastatin was initiated, and computed tomography angiography (CTA) of the chest was ordered. 

**Table 1:** Comparison of initial laboratory findings obtained at the referring institution and repeat testing performed at our institution. Elevated D-dimer levels, transient troponin elevation, leukocytosis with neutrophilia, and moderate anemia were observed during the diagnostic workup.

Test	Initial Laboratory Results	Follow-up Laboratory Results	Reference Range	Interpretation
Troponin T	22.64 ng/mL	—	<0.014 ng/mL	Markedly elevated
Troponin I	0.012 ng/mL	0.010 ng/mL	<0.056 ng/mL	Within normal limits
D-dimer	8,802 ng/mL	4,491 ng/mL	<500 ng/mL	Elevated
White blood cell count	12,670 cells/µL	—	4,000–10,000 cells/µL	Leukocytosis
Neutrophils	Elevated	—	40–70%	Neutrophilia
Hemoglobin	8.3 g/dL	9.4 g/dL	12–16 g/dL	Moderate anemia

**Figure 1:**
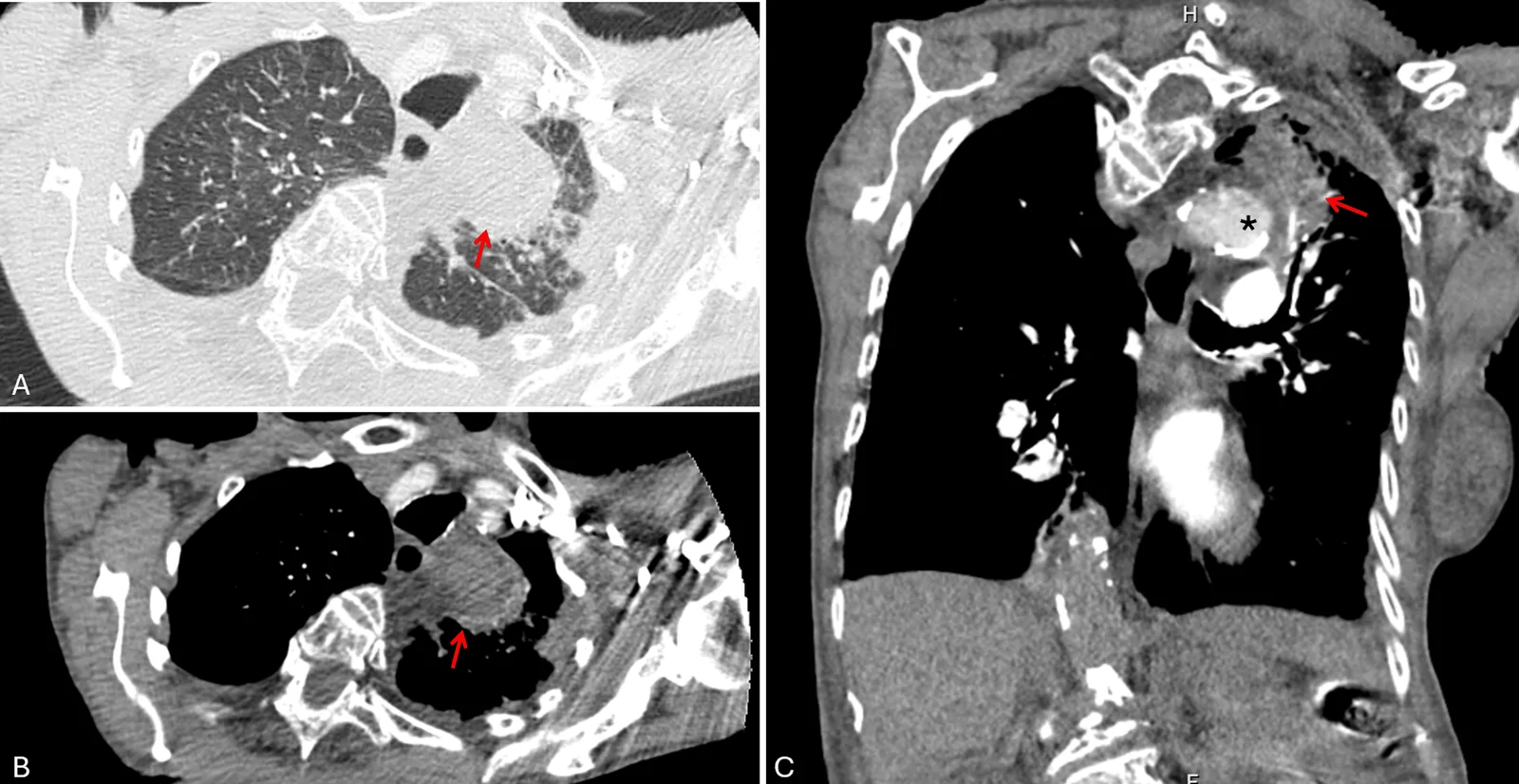
Chest CTA images obtained using a PE protocol. (A) Axial lung window. (B) Axial mediastinal window. (C) Coronal reformatted image. A soft-tissue density lesion (red arrow) is observed in close contact with the aortic arch (*), initially interpreted as a pulmonary mass. CTA: computed tomography angiography, PE: pulmonary embolism.

CTA performed using a PE protocol excluded filling defects within the pulmonary arteries. However, it revealed a 42 × 38 × 70 mm soft-tissue density lesion in the apicoposterior segment of the left upper lobe, in close contact with the aortic arch (Figure 1). In light of this finding, together with a subsequent report of a 4-kg weight loss over the preceding three months and the presence of cutaneous lesions (erythroderma), pulmonary neoplasia with lymphangitic spread was suspected. 

**Figure 2:**
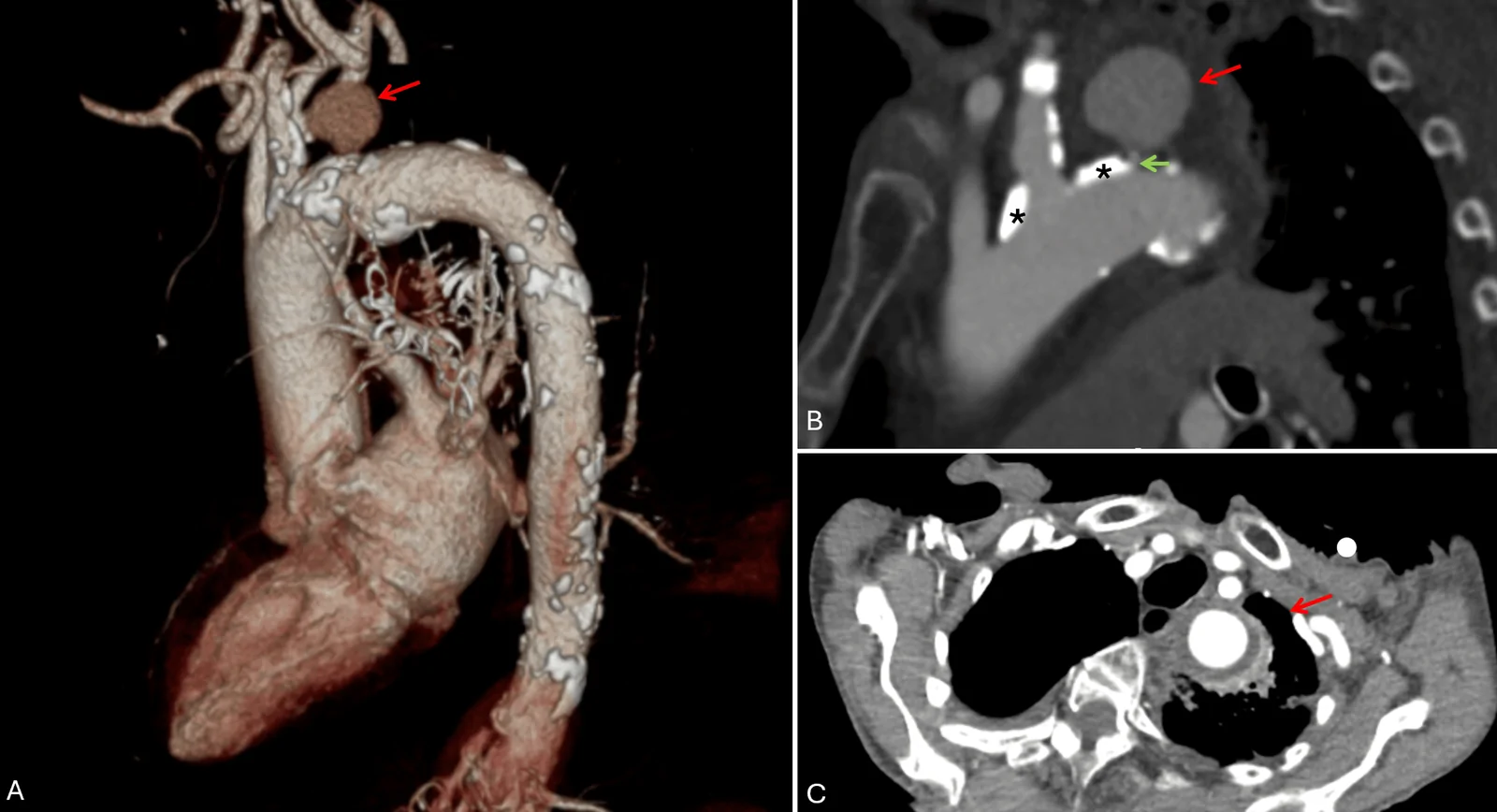
Chest CTA images obtained using an aortic protocol. (A) Three-dimensional reconstruction. (B) Sagittal reformatted image. (C) Axial mediastinal window A saccular outpouching arising from the aortic arch (red arrow) demonstrates the same contrast enhancement as the aortic lumen, consistent with a pseudoaneurysm secondary to a penetrating aortic ulcer. The neck of the pseudoaneurysm is indicated by the green arrow, and adjacent calcified atherosclerotic plaques are marked with asterisks (*). No contrast extravasation into the pleural cavity is identified. CTA: computed tomography angiography.

The patient was evaluated by the Thoracic Surgery service. After assessing the lesion’s location, its anatomical relationship with the aortic arch, and the stability of cardiac biomarkers, the team raised concern for acute aortic syndrome, specifically a penetrating aortic ulcer associated with an intramural hematoma. Consequently, a chest CTA using an aortic protocol was requested. This examination demonstrated a penetrating ulcer with a secondary pseudoaneurysm arising from the aortic arch (Figure 2).

**Figure 3:**
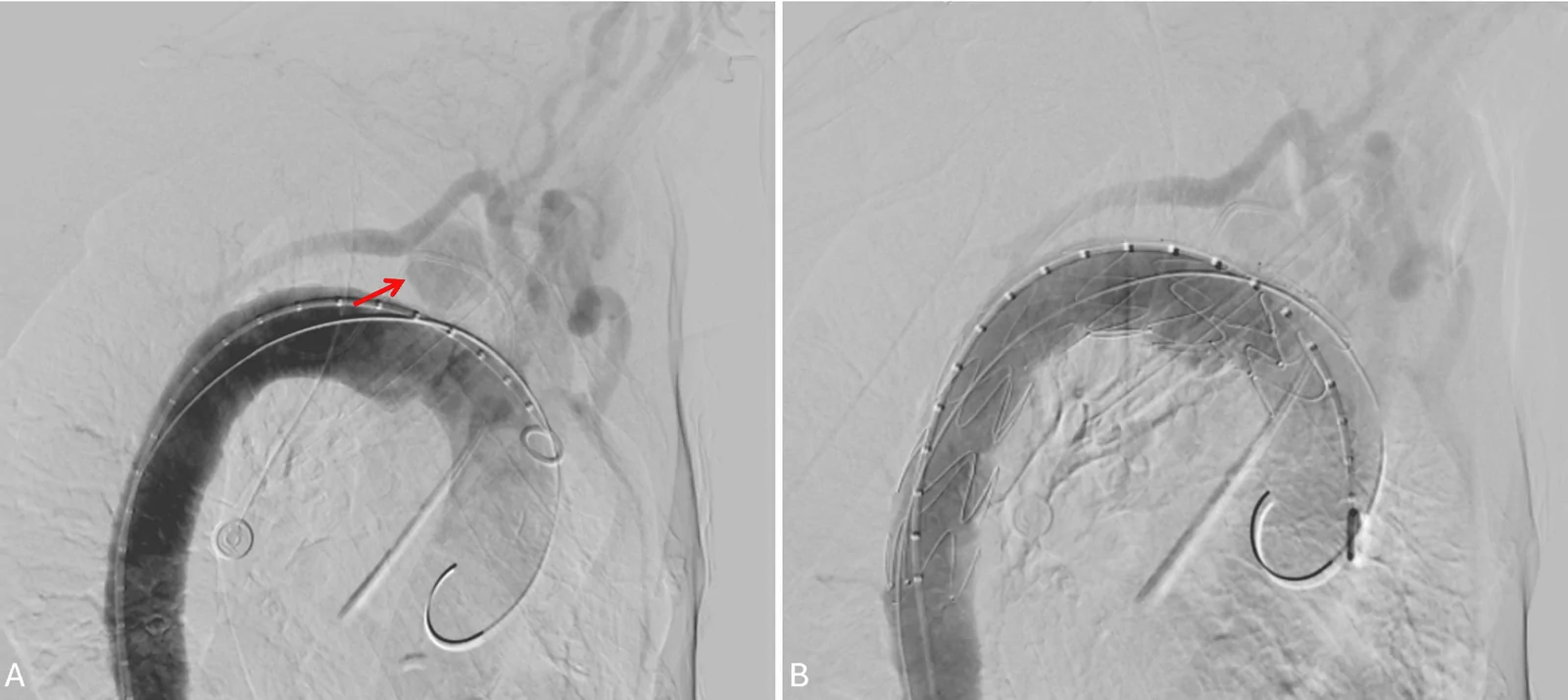
Aortic angiography images. (A) Oblique view before repair. (B) Oblique view after endovascular stent-graft implantation. Panel A demonstrates contrast extravasation through a defect in the aortic arch (red arrow), with complete exclusion of the lesion following endovascular repair.

Given the patient's frailty and the risk of rupture, the Vascular Surgery team opted for thoracic endovascular aortic repair (TEVAR). Surgical exposure of the right common femoral artery and percutaneous access of the left common femoral artery were obtained. Under fluoroscopic guidance and intraoperative angiography, a 30 × 155 mm Zenith Alpha thoracic endovascular graft (Cook Medical, Bloomington, Indiana) was deployed with the proximal landing zone immediately distal to the origin of the left subclavian artery, preserving patency of the supra-aortic branches. Completion angiography demonstrated adequate graft position and patency without stenosis, endoleak, or contrast extravasation. Balloon molding with a CODA balloon was performed to optimize graft apposition after minimal delayed filling of the pseudoaneurysm was observed on the initial completion angiogram. The procedure was technically successful, with no immediate procedural complications (Figure 3).

The postoperative course was uneventful. During hospitalization, the patient was diagnosed with atrial fibrillation. She remained clinically stable and was discharged on apixaban 2.5 mg twice daily for atrial fibrillation and aspirin 100 mg once daily, as recommended by the vascular surgery team following TEVAR, in addition to apixaban 2.5 mg twice daily, aspirin 100 mg once daily, atorvastatin 40 mg once daily, and metoprolol succinate 150 mg once daily, with scheduled outpatient follow-up.

## Discussion

AAS is often regarded as the “great mimicker” of emergency medicine. This case illustrates how its clinical presentation can redirect the diagnostic process toward coronary or neoplastic conditions.

The markedly elevated D-dimer represented the first diagnostic pitfall. Although D-dimer is widely used in the evaluation of pulmonary embolism, its sensitivity for AAS exceeds 95%, albeit with limited specificity [1,7]. The ADvISED study demonstrated that combining clinical risk assessment with D-dimer testing is essential to minimize missed diagnoses of AAS [3]. In this case, the markedly elevated D-dimer level (8,802 ng/mL) should have prompted consideration of acute aortic pathology early in the diagnostic process. Likewise, the initial elevation of troponin, which subsequently normalized, is a recognized finding in acute aortic syndrome. Proposed mechanisms include coronary malperfusion, myocardial oxygen supply-demand mismatch, and myocardial injury related to the systemic inflammatory response and acute hemodynamic stress [1,2]. Consequently, patients may be misdiagnosed with acute coronary syndrome and receive antiplatelet or anticoagulant therapy before acute aortic syndrome is excluded, potentially increasing the risk of bleeding and delaying definitive treatment [1,2].

The most challenging aspect of this case was the identification of a presumed pulmonary mass. The literature describes cases in which ruptured or contained aortic pseudoaneurysms have been mistaken for lung carcinoma because of their mass-like appearance and the presence of constitutional symptoms, including weight loss, fever, and erythroderma, resulting from a systemic inflammatory response [4,5]. Conversely, primary lung tumors have occasionally been misdiagnosed as aortic dissections, underscoring the diagnostic complexity of the mediastinal region [8]. In the present case, the key diagnostic step was the transition to a dedicated aortic imaging protocol, which allowed accurate visualization of the PAU and associated pseudoaneurysm that could not be adequately characterized on the initial pulmonary embolism protocol CTA.

Successful treatment with thoracic endovascular aortic repair in an 87-year-old patient underscores that advanced age and frailty should not preclude appropriate interventional management. According to the European Society for Vascular Surgery (ESVS) guidelines, endovascular repair is the preferred treatment for complicated thoracic aortic disease, offering lower morbidity and mortality than open surgical repair [6].

Recent evidence further supports the use of TEVAR in elderly patients. A systematic review and meta-analysis by Frisiras et al. demonstrated that elective TEVAR in octogenarians is associated with perioperative morbidity and mortality rates comparable to those observed in younger patients, although long-term survival remains lower because of age-related comorbidities. These findings suggest that advanced age alone should not preclude endovascular treatment and that careful patient selection remains the key determinant of favorable outcomes. Our case further illustrates that successful TEVAR can be safely performed even in a frail 87-year-old patient when appropriately indicated [9].

This case highlights the importance of maintaining a high index of suspicion for AAS despite initially misleading clinical and imaging findings, particularly in elderly patients.

## Conclusions

This case highlights the importance of maintaining a high index of suspicion for acute aortic syndrome in elderly patients presenting with chest pain, even when imaging findings initially suggest an alternative diagnosis such as pulmonary malignancy. Comprehensive evaluation of thoracic masses with contrast-enhanced CT is essential, particularly when the lesion is adjacent to the aortic wall, as it allows accurate characterization and may prevent potentially catastrophic invasive procedures such as biopsy. When a thoracic mass is identified near the aortic wall in a patient presenting with acute chest pain and a markedly elevated D-dimer level, dedicated aortic CTA should be considered early in the diagnostic workup. This case underscores the pivotal role of CT in establishing the correct diagnosis, guiding appropriate management, and ultimately improving patient outcomes.
